# The genome sequence of the Tyrrhenian Wall Lizard, *Podarcis tiliguerta* (Gmelin, 1789) (Squamata: Lacertidae)

**DOI:** 10.12688/wellcomeopenres.24853.1

**Published:** 2025-09-08

**Authors:** Nathalie Feiner, Tobias Uller, Daniele Salvi, Joana Meier, Wellcome Sanger Institute Tree of Life Management, Samples and Laboratory team, Wellcome Sanger Institute Scientific Operations: Sequencing Operations, Wellcome Sanger Institute Tree of Life Core Informatics team, Tree of Life Core Informatics collective

**Affiliations:** 1 Max Planck Institute for Evolutionary Biology, Plön, Germany; 2 Department of Biology, Lund University, Lund, Skåne County, Sweden; 3 University of L’Aquila, L’Aquila, Italy; 4 Tree of Life, Wellcome Sanger Institute, Hinxton, England, UK

**Keywords:** Podarcis tiliguerta; Tyrrhenian Wall Lizard; genome sequence; chromosomal; Squamata

## Abstract

We present a genome assembly from an individual female *Podarcis tiliguerta* (Tyrrhenian Wall Lizard; Chordata; Lepidosauria; Squamata; Lacertidae). The assembly contains two haplotypes with total lengths of 1 462.31 megabases and 1 394.94 megabases. Most of haplotype 1 (99.26%) is scaffolded into 20 chromosomal pseudomolecules, including the W and Z sex chromosomes. Most of haplotype 2 (99.2%) is scaffolded into 18 chromosomal pseudomolecules. The mitochondrial genome has also been assembled, with a length of 17.19 kilobases.

## Species taxonomy

Eukaryota; Opisthokonta; Metazoa; Eumetazoa; Bilateria; Deuterostomia; Chordata; Craniata; Vertebrata; Gnathostomata; Teleostomi; Euteleostomi; Sarcopterygii; Dipnotetrapodomorpha; Tetrapoda; Amniota; Sauropsida; Sauria; Lepidosauria; Squamata; Bifurcata; Unidentata; Episquamata; Laterata; Lacertibaenia; Lacertidae; Lacertinae; *Podarcis*; *Podarcis tiliguerta* (Gmelin, 1789) (NCBI:txid65485)

## Background

**Figure 1.**
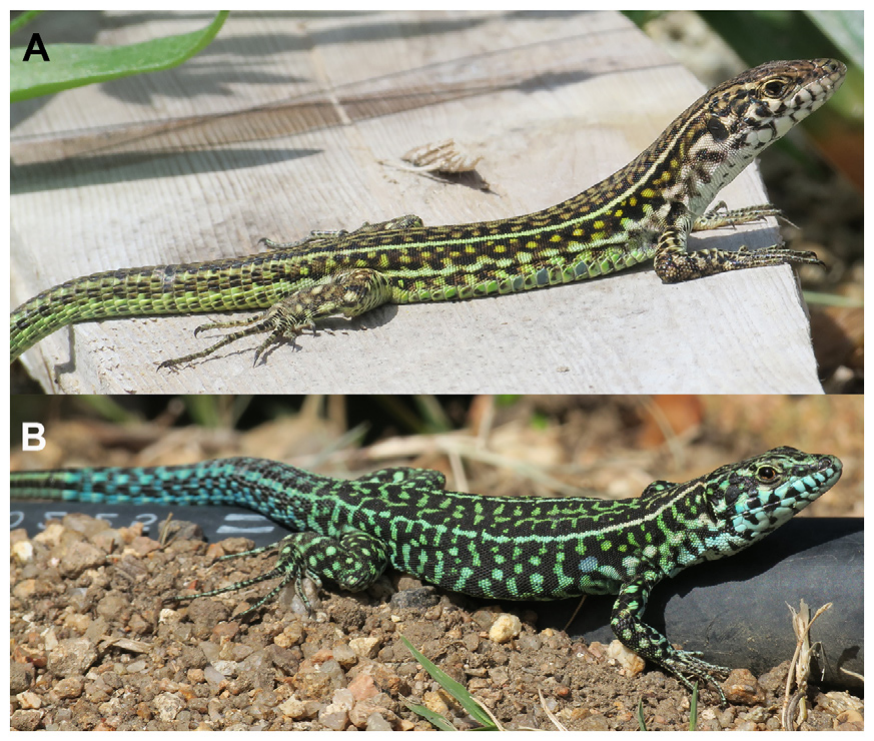
Photograph of the *Podarcis tiliguerta* (rPodTil1) specimen used for genome sequencing.

The Tyrrhenian wall lizard *Podarcis tiliguerta* (Gmelin, 1789; Figure 1) is a diurnal lacertid lizard endemic to Corsica, Sardinia and surrounding islets. It is common throughout the main islands and is only absent in agricultural areas and at high altitudes on Corsica, although it occurs at least up to 1800 m ( Mertens, 1957). More than 10 subspecies have been described, reflecting the large phenotypic diversity found in this species ( Böhme, 1986). *Podarcis tiliguerta* is classified as Least Concern (LC) by the IUCN ( Bowles, 2024).

This species is one of 28 currently described species of the genus *Podarcis*. *P. tiliguerta* is the sister species to the group of *P. lilfordi* and *P. pityusensis* ( Yang *et al.*, 2021), which are endemic to the Balearic islands. The divergence between *P. tiliguerta* and the Balearic species is estimated to have occurred over 10 MYA ( Yang *et al.*, 2021). The lineages of *P. tiliguerta* that inhabit Sardinia and Corsica are highly divergent and further genetic differentiation exist within each of these lineages ( Harris *et al.*, 2005; Rodríguez *et al.*, 2017; Salvi *et al.*, 2017; Senczuk *et al.*, 2019).

The species can be found in a broad variety of habitats, including Mediterranean shrubland, bushy vegetation, grassland, sandy or rocky shores as well as anthropogenic environments. Lizards can be seen active all year round, except at high altitude, but the main activity period is in spring and summer. *P. tiliguerta* is oviparous and reported to lay multiple clutches per year ( Böhme, 1986); clutch size is uncertain.

Tyrrhenian wall lizards exhibit high diversity in coloration and body size and some island populations can differ substantially from populations on the mainland in these characters ( Böhme, 1986). As in most species of *Podarcis*, the sexes differ in appearance with females being generally smaller and showing a pattern that is more dominated by longitudinal stripes.

*P. tiliguerta* is one of three species of the Western Islands group of *Podarcis*. A reference genome of the species *P. lilfordi* is currently available ( Gomez-Garrido *et al.*, 2023), and a reference for the third species of this group, *P. pityusensis* has recently been generated ( Meier *et al.*, 2025). Reference genomes for every species in this group will be a valuable resource for comparative studies above the species level. In addition, we anticipate that the reference genome of *P. tiliguerta* will support ongoing research on the evolutionary history of this species and help to resolve taxonomic uncertainty regarding the status of different lineages.

We present a chromosome-level genome sequence for *Podarcis tiliguerta*, the Tyrrhenian Wall Lizard. The assembly was produced using the Tree of Life pipeline from a specimen collected in Punta Falcone, Sardinia, Italy ( Figure 1). This assembly was generated as part of the Darwin Tree of Life Project, which aims to generate high-quality reference genomes for all named eukaryotic species in Britain and Ireland to support research, conservation, and the sustainable use of biodiversity ( Blaxter *et al.*, 2022).

## Methods

### Sample acquisition

The specimen, an adult female *P. tiliguerta* lizard (specimen ID SAN25001764, ToLID rPodTil1; Figure 1), was collected on September 10 ^th^ 2019 from a site near Punta Falcone on northern Sardinia (latitude: 41.25152; longitude: 9.22911). The specimen was caught by noosing, standard morphometric measurements were taken and the tip of the tail (ca. 2 cm) was collected and preserved in ethanol. The specimen was released again at the site of capture. Field work was conducted under a permit from the Italian Ministry of Environment: PNM-2019-0008130 (MATTM, Ministero dell’Ambiente e della Tutela del Territorio e del Mare, Italy).

### Nucleic acid extraction

Protocols for high molecular weight (HMW) DNA extraction developed at the Wellcome Sanger Institute (WSI) Tree of Life Core Laboratory are available on protocols.io ( Howard *et al.*, 2025). The rPodTil1 sample was weighed and triaged to determine the appropriate extraction protocol. Tissue from the terminal body was homogenised by powermashing using a PowerMasher II tissue disruptor. HMW DNA was extracted using the Automated MagAttract v2 protocol. DNA was sheared into an average fragment size of 12–20 kb following the Megaruptor®3 for LI PacBio protocol. Sheared DNA was purified by manual SPRI (solid-phase reversible immobilisation). The concentration of the sheared and purified DNA was assessed using a Nanodrop spectrophotometer and Qubit Fluorometer using the Qubit dsDNA High Sensitivity Assay kit. Fragment size distribution was evaluated by running the sample on the FemtoPulse system. For this sample, the final post-shearing DNA had a Qubit concentration of 21.0 ng/μL and a yield of 2 730.00 ng.

### PacBio HiFi library preparation and sequencing

Library preparation and sequencing were performed at the WSI Scientific Operations core. Libraries were prepared using the SMRTbell Prep Kit 3.0 (Pacific Biosciences, California, USA), following the manufacturer’s instructions. The kit includes reagents for end repair/A-tailing, adapter ligation, post-ligation SMRTbell bead clean-up, and nuclease treatment. Size selection and clean-up were performed using diluted AMPure PB beads (Pacific Biosciences). DNA concentration was quantified using a Qubit Fluorometer v4.0 (ThermoFisher Scientific) and the Qubit 1X dsDNA HS assay kit. Final library fragment size was assessed with the Agilent Femto Pulse Automated Pulsed Field CE Instrument (Agilent Technologies) using the gDNA 55 kb BAC analysis kit.

The sample was sequenced on a Revio instrument (Pacific Biosciences). The prepared library was normalised to 2 nM, and 15 μL was used for making complexes. Primers were annealed and polymerases bound to generate circularised complexes, following the manufacturer’s instructions. Complexes were purified using 1.2X SMRTbell beads, then diluted to the Revio loading concentration (200–300 pM) and spiked with a Revio sequencing internal control. The sample was sequenced on a Revio 25M SMRT cell. The SMRT Link software (Pacific Biosciences), a web-based workflow manager, was used to configure and monitor the run and to carry out primary and secondary data analysis.

### Hi-C

***Sample preparation and crosslinking***

The Hi-C sample was prepared from 20–50 mg of frozen tissue from the terminal body of the rPodTil1 sample using the Arima-HiC v2 kit (Arima Genomics). Following the manufacturer’s instructions, tissue was fixed and DNA crosslinked using TC buffer to a final formaldehyde concentration of 2%. The tissue was homogenised using the Diagnocine Power Masher-II. Crosslinked DNA was digested with a restriction enzyme master mix, biotinylated, and ligated. Clean-up was performed with SPRISelect beads before library preparation. DNA concentration was measured with the Qubit Fluorometer (Thermo Fisher Scientific) and Qubit HS Assay Kit. The biotinylation percentage was estimated using the Arima-HiC v2 QC beads.

***Hi-C library preparation and sequencing***

Biotinylated DNA constructs were fragmented using a Covaris E220 sonicator and size selected to 400–600 bp using SPRISelect beads. DNA was enriched with Arima-HiC v2 kit Enrichment beads. End repair, A-tailing, and adapter ligation were carried out with the NEBNext Ultra II DNA Library Prep Kit (New England Biolabs), following a modified protocol where library preparation occurs while DNA remains bound to the Enrichment beads. Library amplification was performed using KAPA HiFi HotStart mix and a custom Unique Dual Index (UDI) barcode set (Integrated DNA Technologies). Depending on sample concentration and biotinylation percentage determined at the crosslinking stage, libraries were amplified with 10 to 16 PCR cycles. Post-PCR clean-up was performed with SPRISelect beads. Libraries were quantified using the AccuClear Ultra High Sensitivity dsDNA Standards Assay Kit (Biotium) and a FLUOstar Omega plate reader (BMG Labtech).

Prior to sequencing, libraries were normalised to 10 ng/μL. Normalised libraries were quantified again and equimolar and/or weighted 2.8 nM pools. Pool concentrations were checked using the Agilent 4200 TapeStation (Agilent) with High Sensitivity D500 reagents before sequencing. Sequencing was performed using paired-end 150 bp reads on the Illumina NovaSeq X.

### Genome assembly

Prior to assembly of the PacBio HiFi reads, a database of *k*-mer counts ( *k* = 31) was generated from the filtered reads using FastK. GenomeScope2 ( Ranallo-Benavidez *et al.*, 2020) was used to analyse the *k*-mer frequency distributions, providing estimates of genome size, heterozygosity, and repeat content.

The HiFi reads were assembled using Hifiasm in Hi-C phasing mode ( Cheng *et al.*, 2021; Cheng *et al.*, 2022), producing two haplotypes. Hi-C reads ( Rao *et al.*, 2014) were mapped to the primary contigs using bwa-mem2 ( Vasimuddin *et al.*, 2019). Contigs were further scaffolded with Hi-C data in YaHS ( Zhou *et al.*, 2023), using the --break option for handling potential misassemblies. The scaffolded assemblies were evaluated using Gfastats ( Formenti *et al.*, 2022), BUSCO ( Manni *et al.*, 2021) and MERQURY.FK ( Rhie *et al.*, 2020).

The mitochondrial genome was assembled using MitoHiFi ( Uliano-Silva *et al.*, 2023), which runs MitoFinder ( Allio *et al.*, 2020) and uses these annotations to select the final mitochondrial contig and to ensure the general quality of the sequence.

### Assembly curation

The assembly was decontaminated using the Assembly Screen for Cobionts and Contaminants ( ASCC) pipeline. TreeVal was used to generate the flat files and maps for use in curation. Manual curation was conducted primarily in PretextView and HiGlass ( Kerpedjiev *et al.*, 2018). Scaffolds were visually inspected and corrected as described by Howe *et al*. (2021). The curation process is documented at https://gitlab.com/wtsi-grit/rapid-curation. PretextSnapshot was used to generate a Hi-C contact map of the final assembly.

### Assembly quality assessment

The Merqury.FK tool ( Rhie *et al.*, 2020) was run in a Singularity container ( Kurtzer *et al.*, 2017) to evaluate *k*-mer completeness and assembly quality for both haplotypes using the *k*-mer databases ( *k* = 31) computed prior to genome assembly. The analysis outputs included assembly QV scores and completeness statistics.

The genome was analysed using the BlobToolKit pipeline, a Nextflow implementation of the earlier Snakemake version ( Challis *et al.*, 2020). The pipeline aligns PacBio reads using minimap2 ( Li, 2018) and SAMtools ( Danecek *et al.*, 2021) to generate coverage tracks. It runs BUSCO ( Manni *et al.*, 2021) using lineages identified from the NCBI Taxonomy ( Schoch *et al.*, 2020). For the three domain-level lineages, BUSCO genes are aligned to the UniProt Reference Proteomes database ( Bateman *et al.*, 2023) using DIAMOND blastp ( Buchfink *et al.*, 2021). The genome is divided into chunks based on the density of BUSCO genes from the closest taxonomic lineage, and each chunk is aligned to the UniProt Reference Proteomes database with DIAMOND blastx. Sequences without hits are chunked using seqtk and aligned to the NT database with blastn ( Altschul *et al.*, 1990). The BlobToolKit suite consolidates all outputs into a blobdir for visualisation. The BlobToolKit pipeline was developed using nf-core tooling ( Ewels *et al.*, 2020) and MultiQC ( Ewels *et al.*, 2016), with containerisation through Docker ( Merkel, 2014) and Singularity ( Kurtzer *et al.*, 2017).

## Genome sequence report

### Sequence data

**Figure 2.**
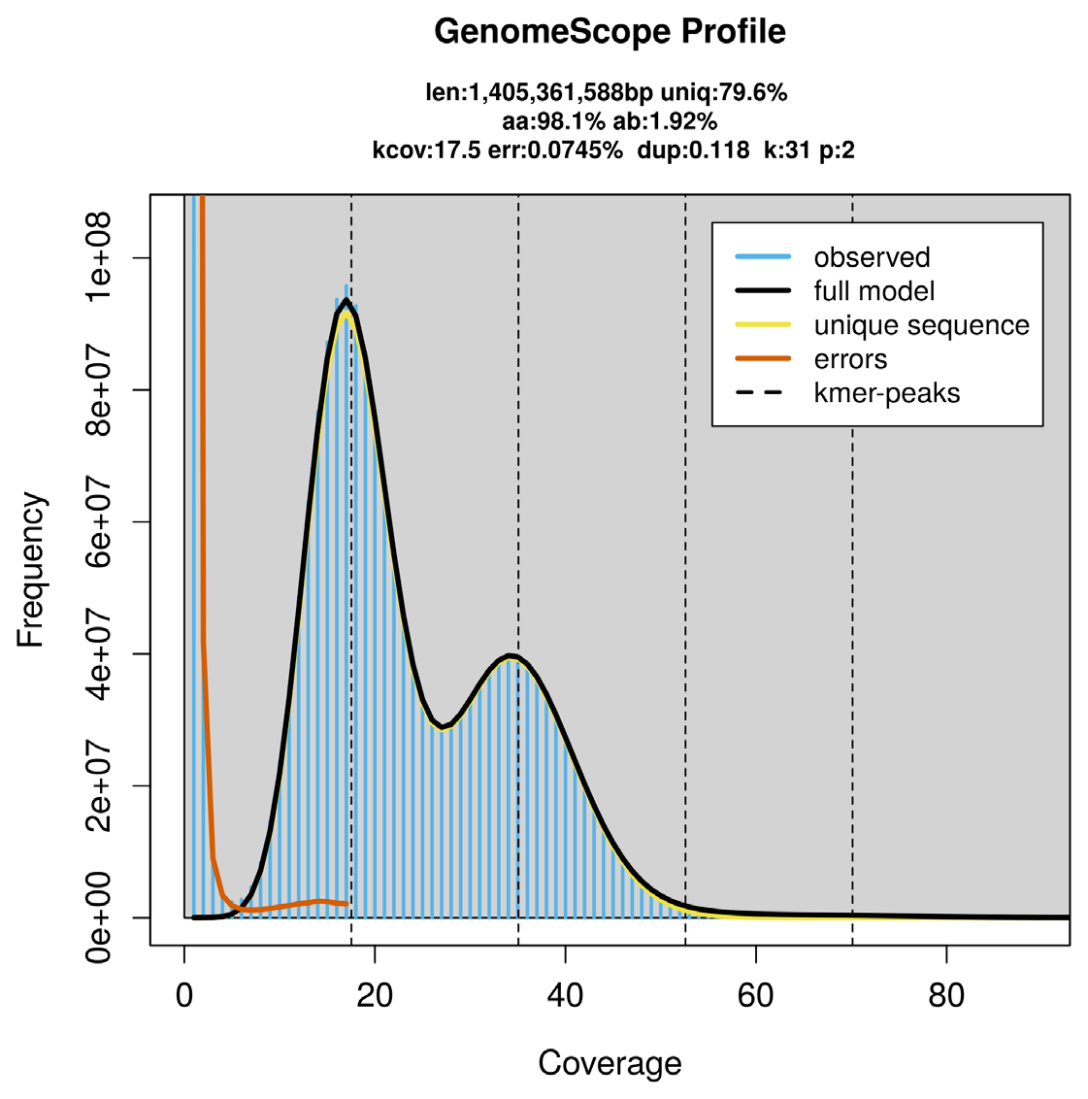
Frequency distribution of *k*-mers generated using GenomeScope2. The plot shows observed and modelled *k*-mer spectra, providing estimates of genome size, heterozygosity, and repeat content based on unassembled sequencing reads.

PacBio sequencing of the *Podarcis tiliguerta* specimen generated 50.67 Gb (gigabases) from 8.30 million reads, which were used to assemble the genome. GenomeScope2.0 analysis estimated the haploid genome size at 1 405.36 Mb, with a heterozygosity of 1.92% and repeat content of 20.45% ( Figure 2). These estimates guided expectations for the assembly. Based on the estimated genome size, the sequencing data provided approximately 15× coverage. Hi-C sequencing produced 221.39 Gb from 1 466.18 million reads, which were used to scaffold the assembly. Table 1 summarises the specimen and sequencing details.

**Table 1.** Specimen and sequencing data for BioProject PRJEB76520.

Platform	PacBio HiFi	Hi-C
**ToLID**	rPodTil1	rPodTil1
**Specimen ID**	SAN25001764	SAN25001764
**BioSample (source individual)**	SAMEA114217799	SAMEA114217799
**BioSample (tissue)**	SAMEA115336781	SAMEA114217803
**Tissue**	terminal body	terminal body
**Instrument**	Revio	Illumina NovaSeq X
**Run accessions**	ERR13265034; ERR13265035	ERR13301067
**Read count total**	8.30 million	1 466.18 million
**Base count total**	50.67 Gb	221.39 Gb

### Assembly statistics

The genome was assembled into two haplotypes using Hi-C phasing. Haplotype 1 was curated to chromosome level, while haplotype 2 was assembled to scaffold level. The final assembly has a total length of 1 462.31 Mb in 257 scaffolds, with 1 024 gaps, and a scaffold N50 of 89.16 Mb ( Table 2).

**Table 2.** Genome assembly statistics.

Metric	Haplotype 1	Haplotype 2
**Assembly name**	rPodTil1.hap1.1	rPodTil1.hap2.2
**Assembly accession**	GCA_965153285.1	GCA_965153305.2
**Assembly level**	chromosome	chromosome
**Span (Mb)**	1 462.31	1 394.94
**Number of chromosomes**	20	18
**Number of contigs**	1 281	1 310
**Contig N50**	2.56 Mb	2.54 Mb
**Number of scaffolds**	257	307
**Scaffold N50**	89.16 Mb	90.89 Mb
**Longest scaffold length (Mb)**	135.08	135.23
**Sex chromosomes**	W and Z	N/A
**Organelles**	Mitochondrion: 17.19 kb	N/A

**Figure 3.**
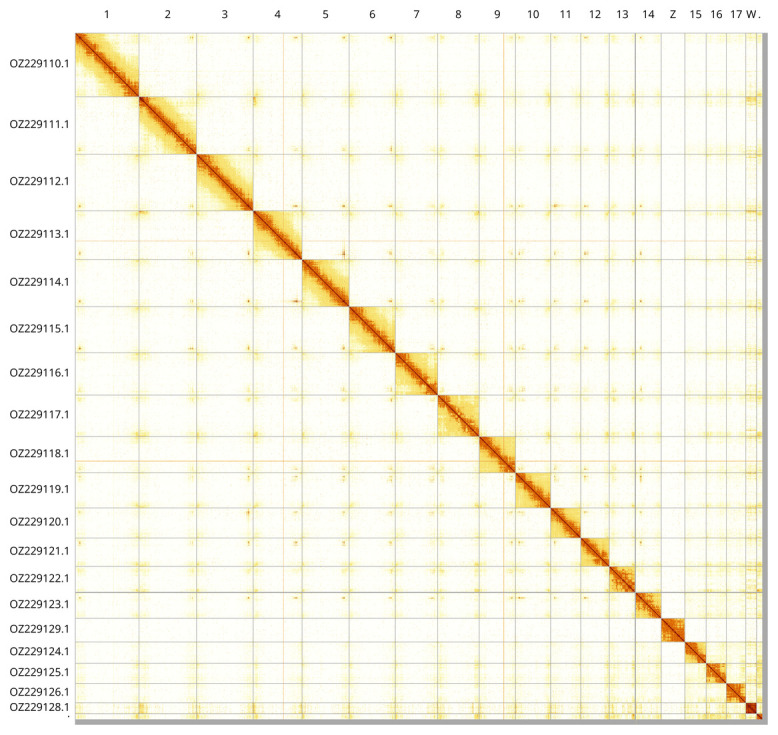
Hi-C contact map of the *Podarcis tiliguerta* genome assembly. Assembled chromosomes are shown in order of size and labelled along the axes. The plot was generated using PretextSnapshot.

Most of the assembly sequence (99.26%) was assigned to 20 chromosomal-level scaffolds, representing 18 autosomes and the W and Z sex chromosomes. These chromosome-level scaffolds, confirmed by Hi-C data, are named according to size ( Figure 3; Table 3). The Z and W chromosomes were identified based on its single-copy status within a diploid assembly and synteny analysis with *Podarcis siculus* (GCA_964188175.1) ( Feiner *et al.*, 2025). The exact order and orientation of the contigs are uncertain in the following regions: chromosome 6 (85 700–89 500 Kbp), chromosome 7 (75 200–1 200 kbp), chromosome 12 (8 200–11 700 kbp), chromosome 14 (9 500 –12 400 kbp). Chromosome 18 is notably small, measuring only 12,718 kbp in the assembled genome.

**Table 3.** Chromosomal pseudomolecules in both haplotypes of the genome assembly of *Calluna vulgaris*, ddCalVulg4.

Haplotype 1				Haplotype 2			
INSDC accession	Name	Length (Mb)	GC%	INSDC accession	Name	Length (Mb)	GC%
OZ229110.1	1	135.08	43.50	OZ229093.1	1	135.23	43.50
OZ229111.1	2	121.46	44	OZ229094.1	2	124.15	44
OZ229112.1	3	119.35	43	OZ229095.1	3	120.05	43
OZ229113.1	4	103.49	43	OZ229096.1	4	103.17	43
OZ229114.1	5	99.17	43.50	OZ229097.1	5	98.92	43.50
OZ229115.1	6	97.79	43.50	OZ229098.1	6	98.44	43.50
OZ229116.1	7	89.16	43	OZ229099.1	7	91.04	43
OZ229117.1	8	87.59	45.50	OZ229100.1	8	87.33	46
OZ229118.1	9	76.80	43.50	OZ229101.1	9	77.10	43.50
OZ229119.1	10	73.99	43.50	OZ229102.1	10	74.60	43.50
OZ229120.1	11	63.69	43.50	OZ229103.1	11	63.77	43.50
OZ229121.1	12	60.19	44	OZ229104.1	12	61.03	43.50
OZ229122.1	13	55.57	45.50	OZ229105.1	13	55.59	45.50
OZ229123.1	14	54.02	45.50	OZ229106.1	14	52.88	45.50
OZ229124.1	15	44.89	46	OZ229107.1	15	44.86	46
OZ229125.1	16	42.81	47	OZ229108.1	16	42.25	47
OZ229126.1	17	41.15	45	OZ229109.1	17	40.65	44.50
OZ229127.1	18	12.75	49	OZ271920.1	18	12.74	49.50
OZ229128.1	W	22.58	45.50				
OZ229129.1	Z	50.01	44.50				

The mitochondrial genome was also assembled. This sequence is included as a contig in the multifasta file of the genome submission and as a standalone record.

**Figure 4.**
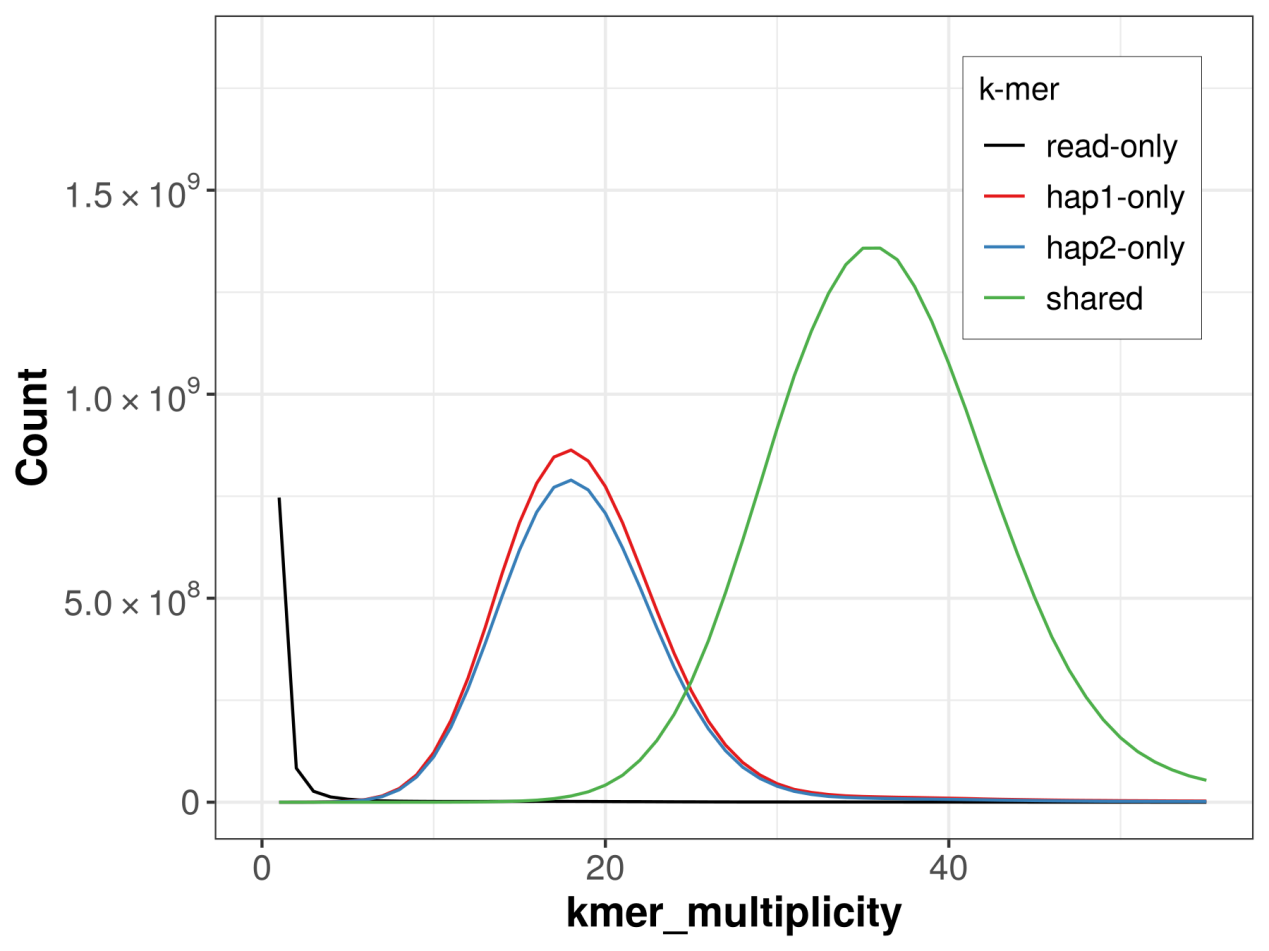
Evaluation of *k*-mer completeness using MerquryFK. This plot illustrates the recovery of *k*-mers from the original read data in the final assemblies. The horizontal axis represents *k*-mer multiplicity, and the vertical axis shows the number of *k*-mers. The black curve represents *k*-mers that appear in the reads but are not assembled. The green curve corresponds to *k*-mers shared by both haplotypes, and the red and blue curves show *k*-mers found only in one of the haplotypes.

For haplotype 1, the estimated QV is 64.6, and for haplotype 2, 64.6. When the two haplotypes are combined, the assembly achieves an estimated QV of 64.6. The *k*-mer completeness is 70.43% for haplotype 1, 67.43% for haplotype 2, and 99.77% for the combined haplotypes ( Figure 4).

**Figure 5.**
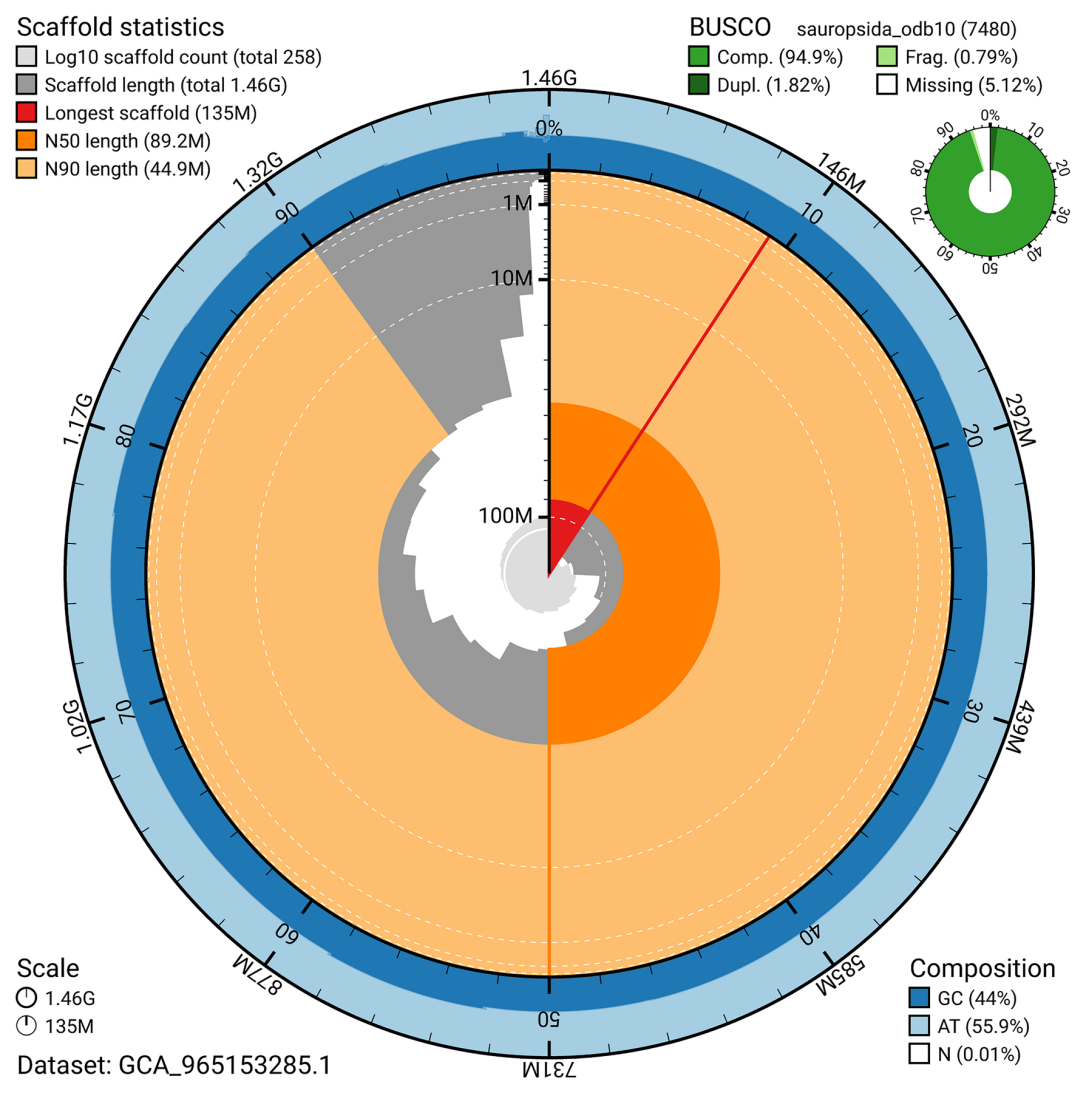
Assembly metrics for rPodTil1.hap1.1. The BlobToolKit snail plot provides an overview of assembly metrics and BUSCO gene completeness. The circumference represents the length of the whole genome sequence, and the main plot is divided into 1 000 bins around the circumference. The outermost blue tracks display the distribution of GC, AT, and N percentages across the bins. Scaffolds are arranged clockwise from longest to shortest and are depicted in dark grey. The longest scaffold is indicated by the red arc, and the deeper orange and pale orange arcs represent the N50 and N90 lengths. A light grey spiral at the centre shows the cumulative scaffold count on a logarithmic scale. A summary of complete, fragmented, duplicated, and missing BUSCO genes in the set is presented at the top right. An interactive version of this figure can be accessed on the BlobToolKit viewer.

**Figure 6.**
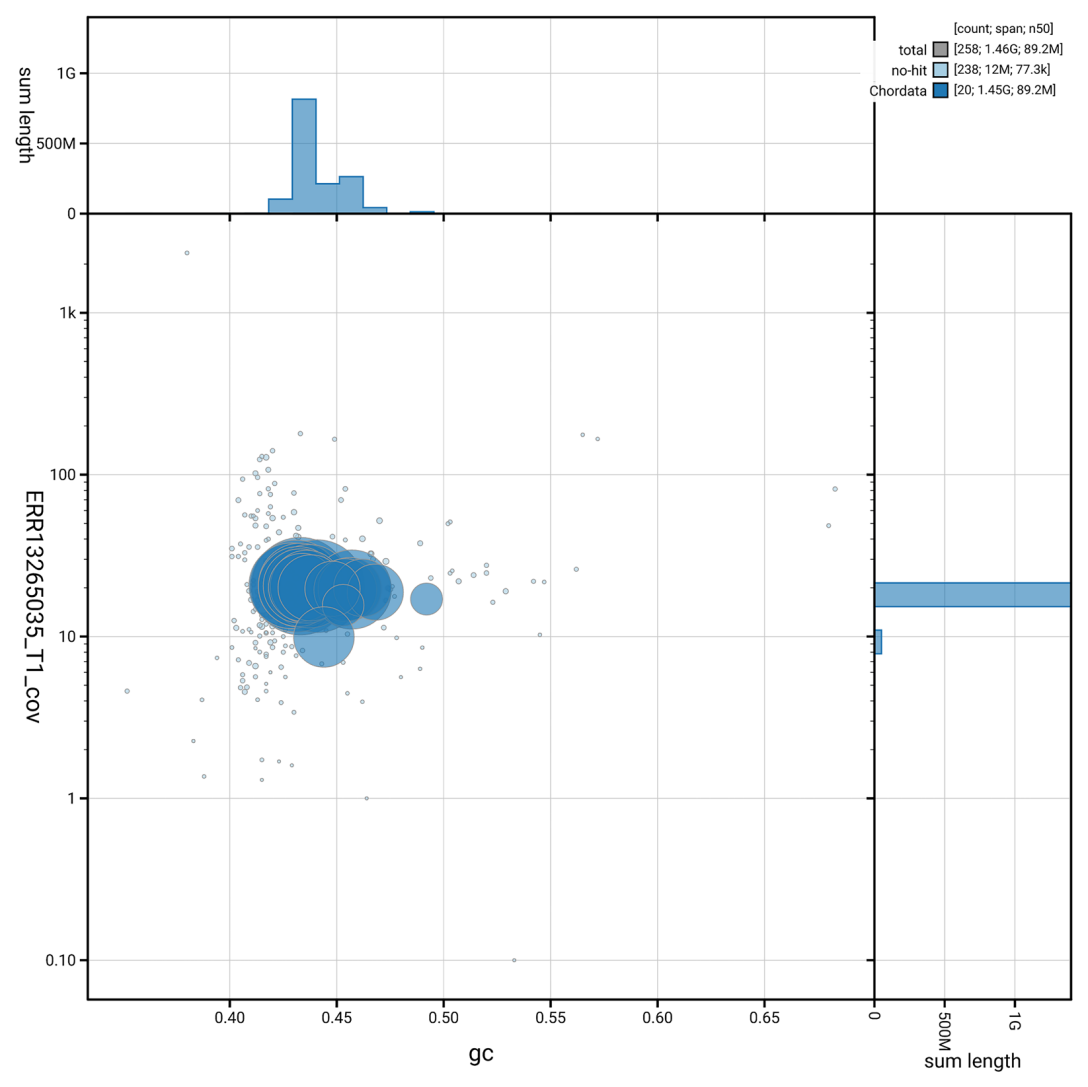
BlobToolKit GC-coverage plot for rPodTil1.hap1.1. Blob plot showing sequence coverage (vertical axis) and GC content (horizontal axis). The circles represent scaffolds, with the size proportional to scaffold length and the colour representing phylum membership. The histograms along the axes display the total length of sequences distributed across different levels of coverage and GC content. An interactive version of this figure is available on the BlobToolKit viewer.

BUSCO analysis using the sauropsida_odb10 reference set ( *n* = 7 480) identified 94.9% of the expected gene set (single = 93.1%, duplicated = 1.8%) for haplotype 1. The snail plot in Figure 5 summarises the scaffold length distribution and other assembly statistics for haplotype 1. The blob plot in Figure 6 shows the distribution of scaffolds by GC proportion and coverage for haplotype 1.

Table 4 lists the assembly metric benchmarks adapted from Rhie *et al.* (2021) the Earth BioGenome Project Report on Assembly Standards September 2024. The EBP metric, calculated for the haplotype 1, is **6.C.Q64**, meeting the recommended reference standard.

**Table 4.** Earth Biogenome Project summary metrics for the *Podarcis tiliguerta* assembly.

Measure	Value	Benchmark
EBP summary (haplotype 1)	6.C.Q64	6.C.Q40
Contig N50 length	2.56 Mb	≥ 1 Mb
Scaffold N50 length	89.16 Mb	= chromosome N50
Consensus quality (QV)	Haplotype 1: 64.6; haplotype 2: 64.6; combined: 64.6	≥ 40
*k*-mer completeness	Haplotype 1: 70.43%; Haplotype 2: 67.43%; combined: 99.77%	≥ 95%
BUSCO	C:94.9% [S:93.1%; D:1.8%]; F:0.8%; M:4.3%; n:7 480	S > 90%; D < 5%
Percentage of assembly assigned to chromosomes	99.26%	≥ 90%

### Wellcome Sanger Institute – Legal and Governance

The materials that have contributed to this genome note have been supplied by a Tree of Life collaborator. The Wellcome Sanger Institute employs a process whereby due diligence is carried out proportionate to the nature of the materials themselves, and the circumstances under which they have been/are to be collected and provided for use. The purpose of this is to address and mitigate any potential legal and/or ethical implications of receipt and use of the materials as part of the research project, and to ensure that in doing so we align with best practice wherever possible.

The overarching areas of consideration are:

- Ethical review of provenance and sourcing of the material
- Legality of collection, transfer and use (national and international)

Each transfer of samples is undertaken according to a Research Collaboration Agreement or Material Transfer Agreement entered into by the Tree of Life collaborator, Genome Research Limited (operating as the Wellcome Sanger Institute) and in some circumstances other Tree of Life collaborators.

## Data Availability

European Nucleotide Archive: Podarcis tiliguerta (Tyrrhenian wall lizard). Accession number PRJEB76520. The genome sequence is released openly for reuse. The *Podarcis tiliguerta* genome sequencing initiative is part of the Sanger Institute Tree of Life Programme (PRJEB43745) and the Vertebrate Genomes Project (PRJNA489243). All raw sequence data and the assembly have been deposited in INSDC databases. The genome will be annotated using available RNA-Seq data and presented through the Ensembl pipeline at the European Bioinformatics Institute. Raw data and assembly accession identifiers are reported in Table 1 and Table 2. Production code used in genome assembly at the WSI Tree of Life is available at https://github.com/sanger-tol. Table 5 lists software versions used in this study.

## Author information

Contributors are listed at the following links:

- Members of the Wellcome Sanger Institute Tree of Life Management, Samples and Laboratory team
- Members of Wellcome Sanger Institute Scientific Operations – Sequencing Operations
- Members of the Wellcome Sanger Institute Tree of Life Core Informatics team
- Members of the Tree of Life Core Informatics collective

## References

[ref-1] Allio R Schomaker-Bastos A Romiguier J : MitoFinder: efficient automated large-scale extraction of mitogenomic data in target enrichment phylogenomics. *Mol Ecol Resour.* 2020;20(4):892–905. 10.1111/1755-0998.13160 32243090 PMC7497042

[ref-2] Altschul SF Gish W Miller W : Basic Local Alignment Search Tool. *J Mol Biol.* 1990;215(3):403–410. 10.1016/S0022-2836(05)80360-2 2231712

[ref-3] Bateman A Martin MJ Orchard S : UniProt: the Universal Protein Knowledgebase in 2023. *Nucleic Acids Res.* 2023;51(D1):D523–D531. 10.1093/nar/gkac1052 36408920 PMC9825514

[ref-4] Blaxter M Mieszkowska N Di Palma F : Sequence locally, think globally: the Darwin Tree of Life Project. *Proc Natl Acad Sci U S A.* 2022;119(4): e2115642118. 10.1073/pnas.2115642118 35042805 PMC8797607

[ref-5] Böhme W : Handbuch der reptilien und amphibien Europas.Vol. 2/II. Aula-Verlag, 1986.

[ref-6] Bowles P : Podarcis tiliguerta.The IUCN Red List of Threatened Species 2024: e.T157293A137862171, 2024. Reference Source

[ref-7] Buchfink B Reuter K Drost HG : Sensitive protein alignments at Tree-of-Life scale using DIAMOND. *Nat Methods.* 2021;18(4):366–368. 10.1038/s41592-021-01101-x 33828273 PMC8026399

[ref-8] Challis R Richards E Rajan J : BlobToolKit – interactive quality assessment of genome assemblies. *G3 (Bethesda).* 2020;10(4):1361–1374. 10.1534/g3.119.400908 32071071 PMC7144090

[ref-9] Cheng H Concepcion GT Feng X : Haplotype-resolved *de novo* assembly using phased assembly graphs with hifiasm. *Nat Methods.* 2021;18(2):170–175. 10.1038/s41592-020-01056-5 33526886 PMC7961889

[ref-10] Cheng H Jarvis ED Fedrigo O : Haplotype-resolved assembly of diploid genomes without parental data. *Nat Biotechnol.* 2022;40(9):1332–1335. 10.1038/s41587-022-01261-x 35332338 PMC9464699

[ref-11] Danecek P Bonfield JK Liddle J : Twelve years of SAMtools and BCFtools. *GigaScience.* 2021;10(2): giab008. 10.1093/gigascience/giab008 33590861 PMC7931819

[ref-12] Ewels P Magnusson M Lundin S : MultiQC: summarize analysis results for multiple tools and samples in a single report. *Bioinformatics.* 2016;32(19):3047–3048. 10.1093/bioinformatics/btw354 27312411 PMC5039924

[ref-13] Ewels PA Peltzer A Fillinger S : The nf-core framework for community-curated bioinformatics pipelines. *Nat Biotechnol.* 2020;38(3):276–278. 10.1038/s41587-020-0439-x 32055031

[ref-14] Feiner N Uller T Salvi D : The genome sequence of the italian wall lizard, *podarcis siculus* (rafinesque-schmaltz, 1810) [version 1; peer review: 1 approved with reservations]. *Wellcome Open Res.* 2025;10:255. 10.12688/wellcomeopenres.24167.1

[ref-15] Formenti G Abueg L Brajuka A : Gfastats: conversion, evaluation and manipulation of genome sequences using assembly graphs. *Bioinformatics.* 2022;38(17):4214–4216. 10.1093/bioinformatics/btac460 35799367 PMC9438950

[ref-16] Gomez-Garrido J Cruz F Alioto TS : Chromosome-level genome assembly of lilford’s wall lizard, *Podarcis lilfordi* (Günther, 1874) from the Balearic Islands (Spain). *DNA Res.* 2023;30(3): dsad008. 10.1093/dnares/dsad008 37137526 PMC10214862

[ref-17] Harris D Pinho C Carretero M : Determination of genetic diversity within the insular lizard *Podarcis tiliguerta* using mtDNA sequence data, with a reassessment of the phylogeny of *Podarcis.* *Amphibia-Reptilia.* 2005;26:401–7. 10.1163/156853805774408676

[ref-18] Howard C Denton A Jackson B : On the path to reference genomes for all biodiversity: lessons learned and laboratory protocols created in the Sanger Tree of Life core laboratory over the first 2000 species. *bioRxiv.* 2025. 10.1101/2025.04.11.648334 PMC12548527 41129326

[ref-19] Howe K Chow W Collins J : Significantly improving the quality of genome assemblies through curation. *GigaScience.* 2021;10(1): giaa153. 10.1093/gigascience/giaa153 33420778 PMC7794651

[ref-20] Kerpedjiev P Abdennur N Lekschas F : HiGlass: web-based visual exploration and analysis of genome interaction maps. *Genome Biol.* 2018;19(1): 125. 10.1186/s13059-018-1486-1 30143029 PMC6109259

[ref-21] Kurtzer GM Sochat V Bauer MW : Singularity: scientific containers for mobility of compute. *PLoS One.* 2017;12(5): e0177459. 10.1371/journal.pone.0177459 28494014 PMC5426675

[ref-22] Li H : Minimap2: pairwise alignment for nucleotide sequences. *Bioinformatics.* 2018;34(18):3094–3100. 10.1093/bioinformatics/bty191 29750242 PMC6137996

[ref-23] Manni M Berkeley MR Seppey M : BUSCO update: novel and streamlined workflows along with broader and deeper phylogenetic coverage for scoring of eukaryotic, prokaryotic, and viral genomes. *Mol Biol Evol.* 2021;38(10):4647–4654. 10.1093/molbev/msab199 34320186 PMC8476166

[ref-24] Meier J Feiner N Uller T : The genome sequence of the ibiza wall lizard, *podarcis pityusensis* (boscá, 1883) [version 1; peer review: 2 approved, 1 approved with reservations]. *Wellcome Open Research.* 2025;10:235. 10.12688/wellcomeopenres.24143.1 40520148 PMC12163369

[ref-25] Merkel D : Docker: lightweight Linux containers for consistent development and deployment. *Linux J.* 2014;2014(239): 2. Reference Source

[ref-26] Mertens R : Unterlagen zu einer "Herpetologia Tyrrhenia" V. Die Amphibien und Reptilien Korsikas. *Senckenbergiana Biologica.* 1957;38(3–4).

[ref-27] Ranallo-Benavidez TR Jaron KS Schatz MC : GenomeScope 2.0 and Smudgeplot for reference-free profiling of polyploid genomes. *Nat Commun.* 2020;11(1): 1432. 10.1038/s41467-020-14998-3 32188846 PMC7080791

[ref-28] Rao SSP Huntley MH Durand NC : A 3D map of the human genome at kilobase resolution reveals principles of chromatin looping. *Cell.* 2014;159(7):1665–1680. 10.1016/j.cell.2014.11.021 25497547 PMC5635824

[ref-29] Rhie A McCarthy SA Fedrigo O : Towards complete and error-free genome assemblies of all vertebrate species. *Nature.* 2021;592(7856):737–746. 10.1038/s41586-021-03451-0 33911273 PMC8081667

[ref-30] Rhie A Walenz BP Koren S : Merqury: reference-free quality, completeness, and phasing assessment for genome assemblies. *Genome Biol.* 2020;21(1): 245. 10.1186/s13059-020-02134-9 32928274 PMC7488777

[ref-31] Rodríguez V Buades JM Brown RP : Evolutionary history of *Podarcis tiliguerta* on Corsica and Sardinia. *BMC Evol Biol.* 2017;17(1): 27. 10.1186/s12862-016-0860-4 28103805 PMC5248522

[ref-32] Salvi D Pinho C Harris DJ : Digging up the roots of an insular hotspot of genetic diversity: decoupled mito-nuclear histories in the evolution of the Corsican-Sardinian endemic lizard *Podarcis tiliguerta.* *BMC Evol Biol.* 2017;17(1): 63. 10.1186/s12862-017-0899-x 28253846 PMC5335832

[ref-33] Schoch CL Ciufo S Domrachev M : NCBI taxonomy: a comprehensive update on curation, resources and tools. *Database (Oxford).* 2020;2020:baaa062. 10.1093/database/baaa062 32761142 PMC7408187

[ref-34] Senczuk G Castiglia R Colangelo P : The role of island physiography in maintaining genetic diversity in the endemic Tyrrhenian wall lizard ( *Podarcis tiliguerta*). *J Zool.* 2019;309(2):140–51. 10.1111/jzo.12705

[ref-35] Uliano-Silva M Ferreira JGRN Krasheninnikova K : MitoHiFi: a python pipeline for mitochondrial genome assembly from PacBio high fidelity reads. *BMC Bioinformatics.* 2023;24(1): 288. 10.1186/s12859-023-05385-y 37464285 PMC10354987

[ref-36] Vasimuddin M Misra S Li H : Efficient architecture-aware acceleration of BWA-MEM for multicore systems.In: *2019 IEEE International Parallel and Distributed Processing Symposium (IPDPS).*IEEE,2019;314–324. 10.1109/IPDPS.2019.00041

[ref-37] Yang W Feiner N Pinho C : Extensive introgression and mosaic genomes of mediterranean endemic lizards. *Nat Commun.* 2021;12(1): 2762. 10.1038/s41467-021-22949-9 33980851 PMC8114931

[ref-38] Zhou C McCarthy SA Durbin R : YaHS: Yet another Hi-C Scaffolding tool. *Bioinformatics.* 2023;39(1): btac808. 10.1093/bioinformatics/btac808 36525368 PMC9848053

