# Supplementary material for: The genome sequence of the Tyrrhenian Wall Lizard, Podarcis tiliguerta (Gmelin, 1789) (Squamata: Lacertidae)
Source: Wellcome Open Res. 2025 Sep 8;10:486. [Version 1] doi: 10.12688/wellcomeopenres.24853.1 (PMC12511862; doi:10.12688/wellcomeopenres.24853.1)
Supplement: Peer review history

# Reviewer report: Reviewer response for version 1

**DOI:** 10.21956/wellcomeopenres.27377.r133864

**Published:** 2025-10-13

**Authors:** Raul Araya Donoso

**Affiliations:** 1 Yale University, New Haven, USA

This is a review of a Data Note submitted to Welcome Open Research by Feiner et al. The authors report the sequencing and assembly of a phased reference genome for the Tyrrhenian Wall Lizard, Podarcis tiliguerta.

 Overall, the note is well written, the methods are adequate for their aims, and the generation of reference genomes for this group of lizards is a big contribution to understanding their evolutionary history.

 I have only minor comments to improve the general delivery of the results.

**Background**

 I want to acknowledge the authors for a well-detailed description of the species and its evolutionary context. This really helps the reader to understand the relevance of the generation of this dataset, which is challenging for “Data Notes”.

**Methods**

 In the Genome Assembly section. The assembly evaluation methods can be removed to avoid redundancy. Since the section that comes after (“Assembly quality assessment”) describes these methods in detail.

 The method used for the identification and naming of chromosomes methods (size and synteny to Podarcis siculus) should be moved to this section.

**Genome Sequence Report**

 Are you currently able to identify which chromosomes are missing from haplotype 2 and present in haplotype 1? If the authors can easily identify it, it would be useful information.

Are sufficient details of methods and materials provided to allow replication by others?

Yes

Is the rationale for creating the dataset(s) clearly described?

Yes

Are the datasets clearly presented in a useable and accessible format?

Yes

Are the protocols appropriate and is the work technically sound?

Yes

Reviewer Expertise:

Evolutionary genomics

I confirm that I have read this submission and believe that I have an appropriate level of expertise to confirm that it is of an acceptable scientific standard, however I have significant reservations, as outlined above.

# Reviewer report: Reviewer response for version 1

**DOI:** 10.21956/wellcomeopenres.27377.r133869

**Published:** 2025-10-09

**Authors:** Andreas Kautt

**Affiliations:** 1 Washington University in St. Louis, St. Louis, USA

The authors generated a high-quality haplotype-phased genome assembly of the Tyrrhenian Wall Lizard endemic to a few islands in the Mediterranean Sea. The assembly was generated using state-of-the-art methods, is characterized by excellent quality metrics, and will provide an important resource for evolutionary genomic and phylogeographic studies. Overall, this is very nice work and I only have two related comments concerning the small size of chr18 and two clarification requests for the authors to consider.

 Main comments:

 - Do the authors have any insights into why chr18 is so small? Have they examined synteny with other available Podarcis assemblies? Did the majority of chr18 end up in haplotype2? It might be worthwhile to investigate this further, as something appears to be missing in haplotype1.

 - Related to the previous point, a BUSCO score of ~95%, while good overall, seems somewhat low for an assembly of this quality and raises the question of whether it is genes on (the full) chr18 that are predominantly the ones that are missing. One way to check this would be to assess whether the BUSCO score of the "primary" assembly (i.e., non-haplotype-resolved hifiasm output) is considerably better than hap1. Another approach would be to check if the missing genes are syntenic in other closely related species, which would support the hypothesis that a chromosome segment is missing from the assembly rather than genes scattered throughout.

 Minor comments:

 - The reference to the Darwin Tree of Life Project seems a bit odd given that this species is endemic to Corsica and Sicily, while the Darwin Tree of Life Project "aims to generate high-quality reference genomes for all named eukaryotic species in Britain and Ireland." I leave it to the authors to determine if it is appropriate to acknowledge the Darwin Tree of Life Project, but maybe they can briefly explain how this assembly fits its scope.

 - I assume hifiasm was run with default settings. Was the expected haploid genome size specified? Please specify.

Are sufficient details of methods and materials provided to allow replication by others?

Yes

Is the rationale for creating the dataset(s) clearly described?

Yes

Are the datasets clearly presented in a useable and accessible format?

Yes

Are the protocols appropriate and is the work technically sound?

Yes

Reviewer Expertise:

Evolutionary genomics

I confirm that I have read this submission and believe that I have an appropriate level of expertise to confirm that it is of an acceptable scientific standard.

# Reviewer report: Reviewer response for version 1

**DOI:** 10.21956/wellcomeopenres.27377.r133867

**Published:** 2025-10-07

**Authors:** Anthony Snead

**Affiliations:** 1 New York University, NY, NY, USA

This work by Feiner and colleagues critical provide a critical genomic resource for understanding macroevolutionary dynamics across wall lizards, while opening the door to population genomic studies using the Tyrrhenian Wall Lizard ( *Podarcis tiliguerta*). They present a chromosome-level haplotype-phased reference grade genome assembly for *Podarcis tiliguerta* with 99.26% of the assembly sequence assigned to 20 chromosomal-level scaffolds representing the 18 autosomes and two sex chromosomes in haplotype 1. The data and assembly quality checks are robust and this promising to be a critical resource for future evolutionary genomic studies. There are only a couple of clarifying comments on specific lines that I have for the authors.

1. The authors state they collected standard morphometric measurements of the specimen. Standard morphometric measurements could differ between labs. I assumed weight and snout to vent length; however, researchers often use calipers to measure limb lengths head width or other measurements. I think clarifying that point and pointing to a table or supplementary file with that data could aid researchers who may want to use that data in the future.
2. The authors mention that the orientation of some contigs are uncertain. I would assume this is due to the manual curation processes, but I think it would help readers to briefly mention why the exact order and orientation of these areas are uncertain.

Overall, I find this to be a robust data resource, and I am excited to see this genomic resource released.

Are sufficient details of methods and materials provided to allow replication by others?

Yes

Is the rationale for creating the dataset(s) clearly described?

Yes

Are the datasets clearly presented in a useable and accessible format?

Yes

Are the protocols appropriate and is the work technically sound?

Yes

Reviewer Expertise:

Population genomics, comparative genomics, evolutionary biology

I confirm that I have read this submission and believe that I have an appropriate level of expertise to confirm that it is of an acceptable scientific standard.
